# RNA editing-based classification of diffuse gliomas: predicting isocitrate dehydrogenase mutation and chromosome 1p/19q codeletion

**DOI:** 10.1186/s12859-019-3236-0

**Published:** 2019-12-24

**Authors:** Sean Chun-Chang Chen, Chung-Ming Lo, Shih-Hua Wang, Emily Chia-Yu Su

**Affiliations:** Graduate Institute of Biomedical Informatics, College of Medical Science and Technology, Taipei Medical University, Taipei, 10675 Taiwan

**Keywords:** RNA editing, Classification, Gliomas, Machine learning, Prognosis, Isocitrate dehydrogenase

## Abstract

**Background:**

Accurate classification of diffuse gliomas, the most common tumors of the central nervous system in adults, is important for appropriate treatment. However, detection of isocitrate dehydrogenase (*IDH*) mutation and chromosome1p/19q codeletion, biomarkers to classify gliomas, is time- and cost-intensive and diagnostic discordance remains an issue. Adenosine to inosine (A-to-I) RNA editing has emerged as a novel cancer prognostic marker, but its value for glioma classification remains largely unexplored. We aim to (1) unravel the relationship between RNA editing and *IDH* mutation and 1p/19q codeletion and (2) predict *IDH* mutation and 1p/19q codeletion status using machine learning algorithms.

**Results:**

By characterizing genome-wide A-to-I RNA editing signatures of 638 gliomas, we found that tumors without *IDH* mutation exhibited higher total editing level compared with those carrying it (Kolmogorov-Smirnov test, *p* < 0.0001). When tumor grade was considered, however, only grade IV tumors without *IDH* mutation exhibited higher total editing level. According to 10-fold cross-validation, support vector machines (SVM) outperformed random forest and AdaBoost (DeLong test, *p* < 0.05). The area under the receiver operating characteristic curve (AUC) of SVM in predicting *IDH* mutation and 1p/19q codeletion were 0.989 and 0.990, respectively. After performing feature selection, AUCs of SVM and AdaBoost in predicting *IDH* mutation were higher than that of random forest (0.985 and 0.983 vs. 0.977; DeLong test, *p* < 0.05), but AUCs of the three algorithms in predicting 1p/19q codeletion were similar (0.976–0.982). Furthermore, 67% of the six continuously misclassified samples by our 1p/19q codeletion prediction models were misclassifications in the original labelling after inspection of 1p/19q status and/or pathology report, highlighting the accuracy and clinical utility of our models.

**Conclusions:**

The study represents the first genome-wide analysis of glioma editome and identifies RNA editing as a novel prognostic biomarker for glioma. Our prediction models provide standardized, accurate, reproducible and objective classification of gliomas. Our models are not only useful in clinical decision-making, but also able to identify editing events that have the potential to serve as biomarkers and therapeutic targets in glioma management and treatment.

## Background

Diffuse gliomas are the most common tumors of the central nervous system (CNS) in adults.

Accurate diagnosis and classification of diffuse gliomas is important for appropriate treatment. Historically diffuse gliomas are categorized predominantly according to histology: astrocytoma (grade II or III), oligodendroglioma (grade II or III), and glioblastoma (grade IV). Patients carrying lower grade gliomas (LGG; grade II or III) have a more-favorable prognosis, while patients with glioblastoma multiforme (GBM; grade IV) tend to have a poor prognosis regardless of recent advances in clinical management [1, 2]. However, histology diagnosis is primarily based on subjective opinion of experienced pathologists; a sample may be graded differently by different pathologists.

In 2016, the World Health Organization (WHO) changed its classification of diffuse gliomas by considering the presence/absence of *isocitrate dehydrogenase* (*IDH*) mutation and chromosome 1p/19q codeletion [3]. A large subset of adult diffuse gliomas now falls into one of the following categories: *IDH* mutation with 1p/19q codeletion (oligodendroglioma), *IDH* mutation without 1p/19q codeletion (most grades II and III astrocytoma), and *IDH* wildtype (most glioblastoma). This new classification has been shown to provide better prognostications. Some studies have found that LGG patients with *IDH* mutation had prolonged overall survival (OS) compared with those carrying wildtype *IDH* [4, 5]. Also, GBM and anaplastic astrocytoma patients who had *IDH* mutation exhibited improved progression-free survival and OS compared with those without *IDH* mutation [6]. Furthermore, patients with both *IDH* mutation and 1p/19q codeletion had increased OS compared with those with only *IDH* mutation [7]. Therefore, identification of the status of *IDH* mutation and 1p/19q codeletion is essential in clinical practice. However, the identification process is time- and cost-intensive and diagnostic discordance remains an issue. For example, immunohistochemistry (IHC) is a common method to detect *IDH* mutation and requires antibodies to recognize mutations. However, IHC fails to detect less common *IDH* mutations and the concordance rate between IHC and Sanger sequencing was estimated to range 88 to 99% [8]. Similarly, fluorescent in situ hybridization (FISH) is widely used in hospitals to detect 1p/19q status, but confirmation from experienced pathologist is needed [9, 10]. Taken together, a single method which provides standardized, accurate and objective prediction of *IDH* mutation and 1p/19q codeletion is warranted.

Recent advance in high throughput molecular profiling (both sequencing and array-based) has promoted the exploration of genome-wide changes during carcinogenesis. Large-scale molecular data and machine learning algorithms has enabled more objective diagnostics. For example, several studies have used DNA methylation data to cluster/classify brain tumors. Ceccarelli et al. [11] identified the association between DNA methylation and the status of 1p/19q codeletion through unsupervised clustering of DNA methylation patterns. *IDH* mutant gliomas were clustered into three groups: (1) presence of 1p/19q codeletion; (2) absence of 1p/19q codeletion and low global DNA methylation; and (3) absence of 1p/19q codeletion and high global DNA methylation. However, the authors did not develop a method capable of predicting *IDH* mutation and 1p/19q codeletion, which limits the clinical utility of DNA methylation. Capper et al. [12] developed a random forest-based classifier to classify approximately 100 CNS tumor types based on DNA methylation patterns. However, DNA methylation-based classification is not clinically practical at present because of the cost and it provides little hint on the identification of driver events during tumor development and progression.

Compared with DNA methylation array, RNA sequencing (RNA-Seq) is cost-effective and provides more hints on the identification of tumor driver events. RNA-Seq data can be used to identify events that could cause tumor development and progression, including single nucleotide variation, gene expression alteration, alternative isoforms, gene fusion, and RNA editing events. Recently, Wang et al. used gene expression data to predict 1p/19q codeletion status with high accuracy [10], highlighting the potential of RNA-related features to serve as prognostic markers for gliomas.

RNA editing, converting nucleotides at the RNA level, increases transcriptome diversity and alters microRNA regulation [13]. The most common type of RNA editing in human is adenosine to inosine (A-to-I) editing, which is catalyzed by the adenosine deaminase acting on RNA (ADAR) enzyme family [14]. Inosine is recognized as guanosine (G) by the cellular machinery, resulting in A-to-G mutation (when comparing edited reads to genome sequence). Recent studies have highlighted a link between RNA editing and tumor development and progression [15]. Choudhury et al. [16] reported a negative correlation between the editing level of miR-376a-5p and glioma tumor volume. The authors found that reduced editing of miR-376a-5p was associated with more aggressive glioblastoma and poor prognosis. Tomaselli et al. [17] reported that reduced editing of miR-222/221 and miR-21 precursors led to cell proliferation and migration in glioblastoma. However, whether genome-wide RNA editing signature is a marker for glioma classification remains largely unexamined.

In this study, we aimed to (1) unravel the relationship between RNA editing and *IDH* mutation and 1p/19q codeletion and (2) develop models which provide standardized, accurate and objective prediction of *IDH* mutation and chromosome 1p/19q codeletion using RNA editing signature. Three supervised learning algorithms including support vector machines (SVM), random forest (RF) and AdaBoost (AB) were used. We also performed feature selection to avoid overfitting and possibly improve prediction performance. RNA editing events that contribute most to the prediction have the potential to serve as biomarkers and therapeutic targets in glioma management and treatment.

## Results

### Sample characteristics

From The Cancer Genome Atlas (TCGA) glioma cohort, we selected tumors that have both RNA-Seq bam files and annotation of *IDH* mutation and 1p/19q codeletion available, resulting in 638 samples [496 low grade glioma (LGG) and 142 glioblastoma multiforme (GBM)]. Samples were classified into three groups based on the status of *IDH* mutation and 1p/19q codeletion (Table 1): (1) IDH wt: samples without *IDH* mutation; (2) IDH mut-codel: samples with both *IDH* mutation and 1p/19q codeletion; and (3) IDH mut-non-codel: samples with only *IDH* mutation (no 1p/19q codeletion). More than half of IDH wt samples were grade IV tumors and classified as GBM. On the contrary, almost all *IDH* mutant tumors (IDH mut-codel and IDH mut-non-codel) belong to LGG. Moreover, the vast majority of IDH mut-codel samples were classified as oligodendroglioma, while more than half of IDH mut-non-codel samples belong to astrocytoma.

**Table 1:** Histology and grade information of the 638 glioma tumors

	IDH wt	IDH mut-codel	IDH mut-non-codel
Cases	225	163	250
Histology			
Astrocytoma	56 (24.9%)	5 (3.1%)	126 (50.4%)
Glioblastoma	133 (59.1%)	0	9 (3.6%)
Oligoastrocytoma	15 (6.7%)	36 (22.1%)	74 (29.6%)
Oligodendroglioma	21 (9.3%)	122 (74.8%)	40 (16.0%)
Unknown	0	0	1 (0.4%)
Grade			
G2	20 (8.9%)	91 (55.8%)	127 (50.8%)
G3	72 (32.0%)	72 (44.2%)	112 (44.8%)
G4	127 (56.4%)	0	8 (3.2%)
Unknown	6 (2.7%)	0	3 (1.2%)

*IDH wt* Samples with wildtype *isocitrate dehydrogenase* (*IDH*), *IDH mut-codel* Samples with both *IDH* mutation and chromosome 1p/19q codeletion, *IDH mut-non-codel* Samples with only *IDH* mutation (no 1p/19q codeletion)

### Identification of A-to-I RNA editing events

We downloaded 638 RNA-Seq bam files from Genomic Data Commons [18]. For each sample we characterized A-to-I editing events on sites reported in REDIportal [19], currently the most comprehensive A-to-I editing database. Among approximately 4.5 million sites in REDIportal, more than 100 thousand sites have at least one editing event in at least one of the 638 samples. To focus on sites that have better discriminative power for classifying gliomas, we removed sites that (1) did not have enough read coverage (< 10 reads); (2) were not edited in > 75% samples; or (3) have small editing variability among samples (see Methods). Finally, the above criteria resulted in 10,642 sites.

**Fig. 1:**
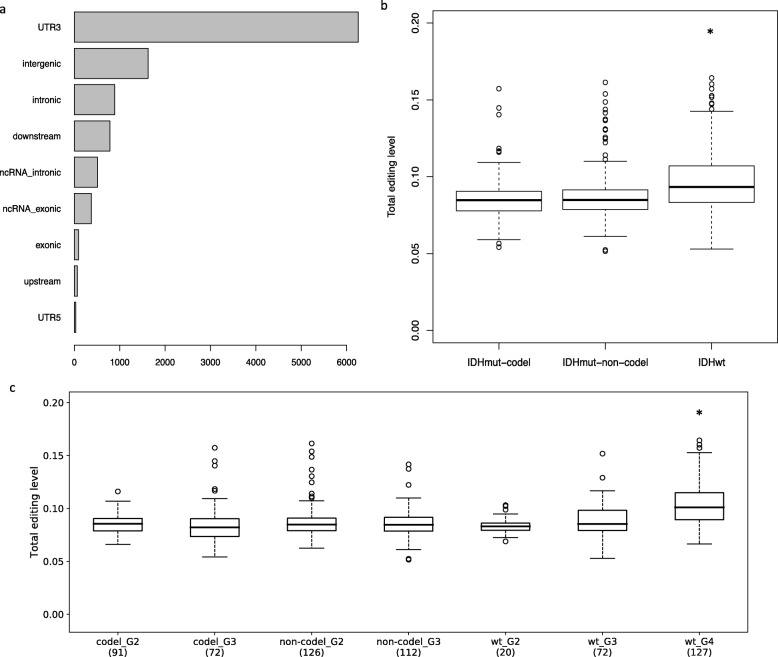
Genic location and editing level of glioma editome. **a** Genic location of 10,642 editing sites using ANNOVAR (RefSeq gene annotation). Sites are located in one of the following ANNOVAR-defined categories: UTR3 (overlaps a 3′ untranslated region), intergenic (in intergenic region), intronic (overlaps an intron), downstream (overlaps 1-kb region downstream of transcription end site), ncRNA (overlaps a transcript without coding annotation in the gene definition), exonic (overlaps a coding), upstream (overlaps 1-kb region upstream of transcription start site), and UTR5 (overlaps a 5′ untranslated region). **b** Total editing level of three glioma subtypes. IDH wt: samples with wildtype isocitrate dehydrogenase (*IDH*); IDH mut-codel: samples with both *IDH* mutation and chromosome 1p/19q codeletion; IDH mut-non-codel: samples with only *IDH* mutation (no 1p/19q codeletion). **c** Total editing level of glioma subtypes considering tumor grade. G2/G3/G4: grade II, III and IV. Asterisk represents statistical difference between subtypes (Kolmogorov-Smirnov test, *p* < 0.0001)

We annotated genic location of the 10,642 sites using ANNOVAR [20] and found that the majority of sites located in 3′ untranslated regions (3’UTR), followed by intergenic and intronic regions (Fig. 1a). To examine the relationship between RNA editing and *IDH* mutation and 1p/19q codeletion status, we calculated total editing level of each sample by considering reads covering the 10,642 sites [total editing level = total (edited G) / total (unedited A+ edited G)]. We found that IDH wt samples, on average, had higher total editing level than IDH mut-non-codel and IDH mut-codel samples (Fig. 1b; Kolmogorov-Smirnov test, *p* < 0.0001). When tumor grade was considered, however, only grade IV tumors with wildtype *IDH* exhibited significantly higher total editing level (Fig. 1c). Our results support the idea that RNA editing has the potential to classify gliomas. Next, we developed models to classify gliomas by predicting the status of *IDH* mutation and 1p/19q codeletion.

### Prediction performance for *IDH* mutation and 1p/19q codeletion

**Fig. 2:**
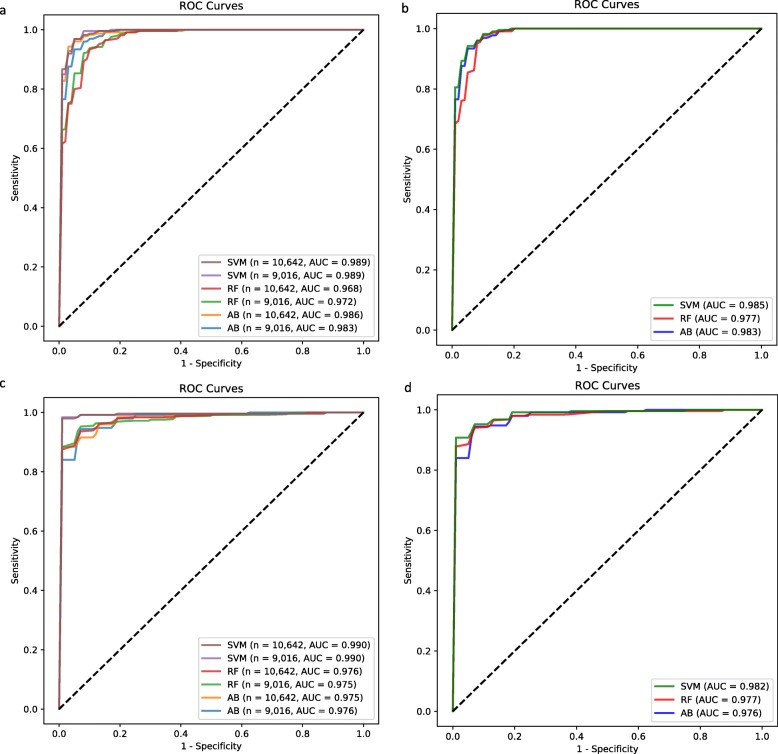
ROC plots for SVM, random forest, and AdaBoost with different number of editing sites. **a** Full models (10,642 and 9016 sites) for predicting *isocitrate dehydrogenase* (*IDH*) mutation. **b** Feature selected models for *IDH* mutation. **c** Full models for predicting chromosome 1p/19q codeletion **d** Feature selected models for 1p/19q codeletion. AB: AdaBoost; RF: random forest; SVM: support vector machines

We first applied three supervised learning algorithms (SVM, RF and AB) to predict the mutation status of *IDH* using RNA editing signatures of the 10,642 sites. Ten-fold cross-validation was applied for generalization of our models and to derive a more accurate estimate of prediction performance. SVM and AB achieved better prediction performance than RF in terms of specificity (SPE) (0.920 and 0.916 vs. 0.764) (Table 2) and the area under the ROC curve (AUC) (0.989 and 0.986 vs. 0.968; DeLong test, *p* < 10^− 4^) (Fig. 2a). To rule out DNA changes misidentified as RNA editing events, for each sample we excluded editing events overlapping with sample-specific somatic mutations or germline variants. However, this approach is not feasible in clinical practice because identification of germline variants is time- and cost-intensive. To make our model more practical, we removed all editing sites that overlap with known variants in the public databases (See Methods). This procedure resulted in 9016 sites and their editing signatures were used to predict the mutation status of *IDH*. The performance of 10,642 sites and 9016 sites were virtually the same (Table 2 and Fig. 2a), suggesting the robustness of our approach.

**Table 2:** Prediction performance for *IDH* mutation

Number of sites	SVM			Random forest			AdaBoost		
	ACC	SEN	SPE	ACC	SEN	SPE	ACC	SEN	SPE
10,642	0.955	0.973	0.920	0.895	0.966	0.764	0.953	0.973	0.916
9016	0.961	0.976	0.933	0.903	0.971	0.778	0.937	0.964	0.889
FS	0.948	0.964	0.920	0.920	0.961	0.844	0.937	0.971	0.876

*FS* Sites selected within each fold using feature importance, *ACC* Accuracy, *SEN* Sensitivity, *SPE* Specificity, *AUC* Area under the receiver operating characteristics curve

Next, to avoid overfitting of SVM and possibly improve prediction performance of RF, we tried to reduce the number of sites used in the models by performing feature selection.

Each algorithm selected a number of sites based on their importance within each cross-validation fold (137~173 sites for SVM, 163~186 sites for RF, and 45~50 sites for AB). Similar to the full models, the feature selected SVM and AB had higher AUCs (0.985 and 0.983, respectively) compared with the feature selected RF (0.977) (DeLong test, *p* = 0.01). Notably, the AUC of the feature selected RF was slightly increased compared with the full models (0.968 and 0.972 for 10,642 sites and 9016 sites, respectively) (DeLong test, *p* = 0.049), probably due to the removal of noise data points. However, for SVM and AB the performance was similar between feature selected and full models (Table 2 and Fig. 2b).

For the prediction of 1p/19q codeletion, SVM outperformed RF and AB in the full models (AUC: 0.990 vs. 0.976 and 0.975; DeLong test, *p* < 0.001) (Table 3 and Fig. 2c). Feature selection resulted in 166~273 sites in SVM, 196~211 sites in RF, and 45~49 sites in AB. The three feature selected classifiers performed similarly (Table 3 and Fig. 2d), but AUC of the feature selected SVM slightly decreased compared with full models (0.982 vs. 0.990; DeLong test, *p* = 0.004).

**Table 3:** Prediction performance for 1p/19q codeletion

Number of sites	SVM			Random forest			AdaBoost		
	ACC	SEN	SPE	ACC	SEN	SPE	ACC	SEN	SPE
10,642	0.971	0.963	0.976	0.913	0.822	0.972	0.939	0.920	0.952
9016	0.983	0.988	0.980	0.918	0.853	0.960	0.932	0.920	0.940
FS	0.937	0.914	0.952	0.930	0.908	0.944	0.932	0.920	0.940

*FS* Sites selected within each fold using feature importance, *ACC* Accuracy, *SEN* Sensitivity, *SPE* Specificity, *AUC* Area under the receiver operating characteristics curve

### RNA editing signatures of sites used in the prediction models

**Fig. 3:**
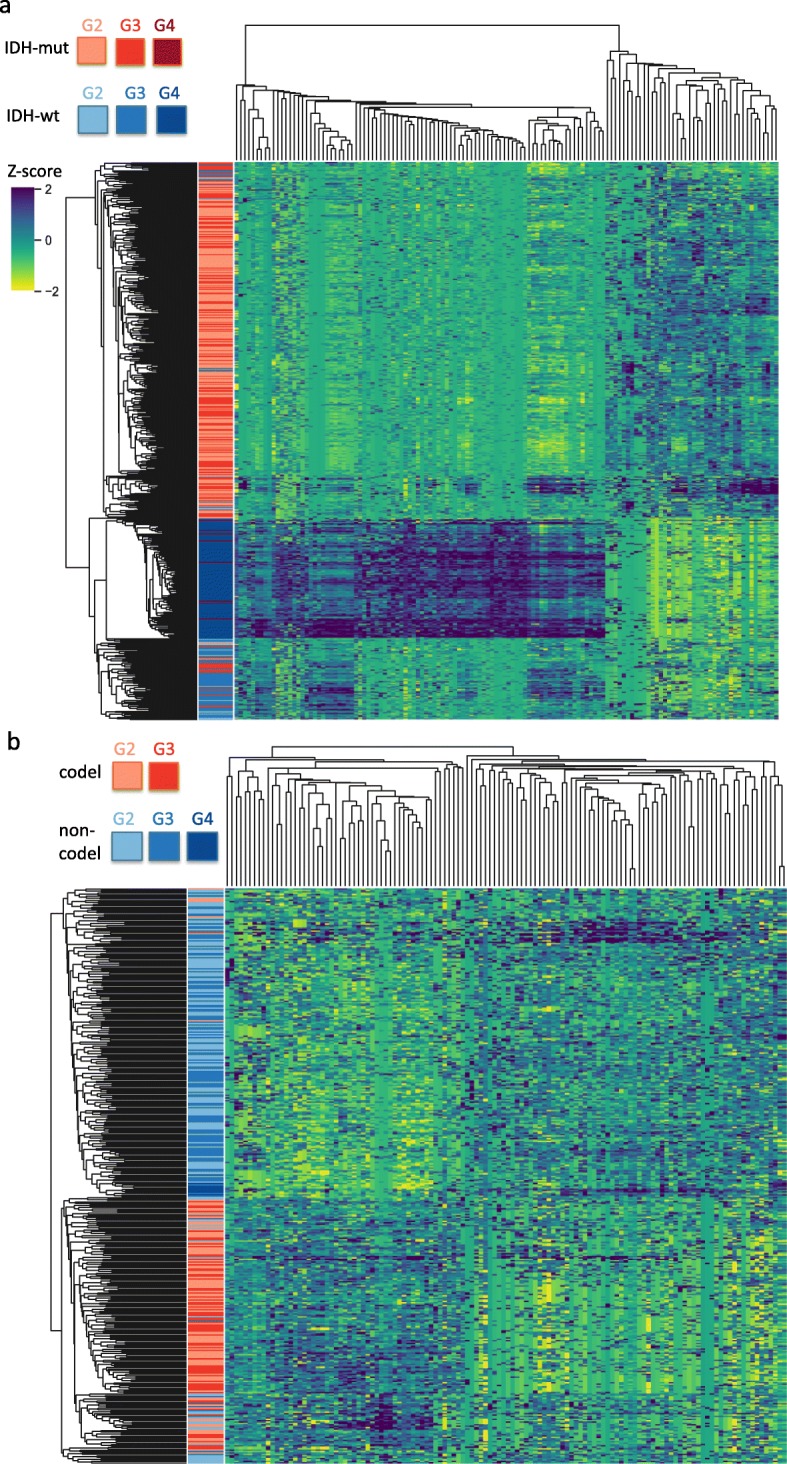
Pan-glioma RNA editing signatures. **a** Heatmap of RNA editing level of the 132 sites repeated selected in the *IDH* mutation classifiers (random forest). Columns represent z-score of RNA editing level of 132 sites sorted by hierarchical clustering. Rows represent 638 TCGA glioma samples sorted by hierarchical clustering. *IDH* mutation status (red: mutant; blue: wildtype) and grade information (G2/G3/G4: grade II, III and IV) of each sample is labeled. **b** Heatmap of z-score of RNA editing level of the 124 sites repeated selected in the 1p/19q codeletion classifiers (random forest). Columns represent RNA editing level of 124 sites sorted by hierarchical clustering. Rows represent 413 TCGA glioma samples (carrying *IDH* mutation) sorted by hierarchical clustering. 1p/19q codeletion status (red: codeletion / blue: non-codeletion) of each sample is labeled

To get a better idea about how glioma samples clustered together using the selected editing sites, we performed hierarchical clustering of editing signatures of sites that were repeatedly selected (at least 5 times) in RF classifiers (132 and 124 sites for *IDH* and 1p/19q codeletion, respectively). Figure 3 shows blocks of editing signatures and these blocks corresponded well to the status of *IDH* and 1p/19q codeletion. Figure 3a reveals sites more heavily edited in *IDH* wildtype (especially GBM) samples as well as sites more heavily edited in *IDH* mutant samples. Figure 3b reveals sites more heavily edited in 1p/19q codeletion samples and also sites more heavily edited in 1p/19q non-codeletion samples.

We next examined the functional and locational enrichment of these repeatedly selected sites. We performed gene enrichment analyses using The Database for Annotation, Visualization and Integrated Discovery (DAVID) v6.8 [21, 22] with the 9016 editing sites as the background. No functional enrichment was detected with FDR < 0.05. For the locational enrichment, we perform hypergeometric test and focused on the top five categories: UTR3, intergenic, intronic, downstream, and ncRNA_intronic. For *IDH* (132 sites), we found enrichment in UTR3 (*p* < 0.02) and depletion in intergenic (*p* < 0.01) and intronic (*p* = 0.02) regions. For 1p/19q codeletion (124 sites), enrichment in ncRNA_intronic (*p* = 0.02) and depletion in UTR3 (*p* = 0.01) and intronic (*p* = 0.01) were detected.

### Examination of the continuously misclassified samples

We also examined the samples continuously misclassified by our models. For *IDH* prediction, 13 samples were continuously misclassified. The misclassification is likely due to heterogeneity and the low number of cases in certain subtypes. For example, three of the 13 misclassified samples are GBM with *IDH* mutation. However, only nine of the 413 samples with *IDH* mutation belong to GBM. For 1p/19q codeletion, six samples were continuously misclassified. Remarkably, 67% of them were misclassifications in the original labelling after inspection of 1p/19q status and/or pathology report, demonstrating the accuracy and clinical utility of our models.

## Discussion

This study represents the first genome-wide RNA editing analysis to date of adult diffuse gliomas. Our analysis demonstrates that RNA editing signature has crucial biological and clinical relevance. Using editing signatures of less than 200 sites, our models achieved high accuracy of predicting *IDH* mutation and 1p/19q codeletion. Compared with the IHC and FISH methods, our models provide more objective diagnostics and avoid labelling error. Four of the six continuously misclassified samples by our 1p/19q codeletion prediction models were misclassifications in the original labelling after inspection of 1p/19q status and/or pathology report, highlighting the accuracy and clinical utility of our models.

**Fig. 4:**
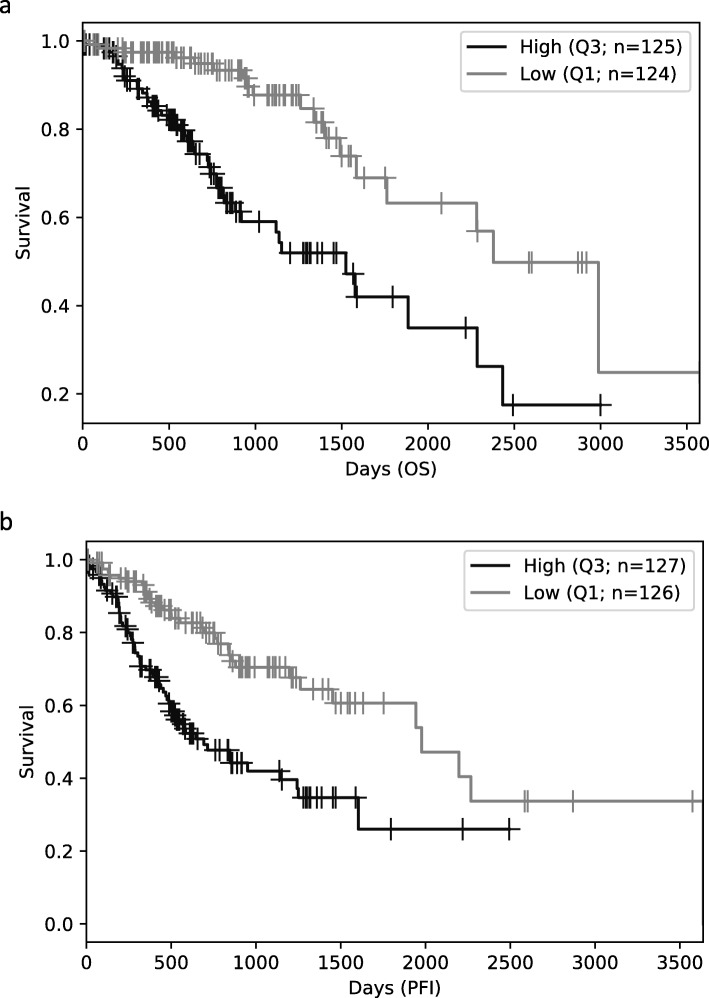
Kaplan-Meier survival curves for LGG samples with different editing level at site chr6:159,679,878. **a** Overall survival (OS) **b** Progression free interval (PFI). This site located on the 3’UTR of the gene *mitochondria-localized manganese superoxide dismutase* (*MnSOD/SOD2*). High (Q3): top 25% samples (with higher editing level). Low (Q1): bottom 25% samples (with lower editing level). Seven and three samples, respectively, were not included in the OS and PFI plots because of lack of data or more than 10 years

Compared with DNA methylation-based classification, our method has some advantages. First, our model is more cost-effective. RNA-Seq has become indispensable in biological research because it generates large amount of data useful for many applications. Currently, the cost of RNA-Seq can be as low as ~$200 per sample, whereas the cost of FISH and Illumina DNA methylation array is ~$340 [10]. With the increasing sequencing output of the Illumina platform, the cost of RNA-Seq will likely be further reduced with time. Additionally, a cost-effective RNA-Seq protocol was proposed recently, which greatly reduced the cost of sample preparation and sequencing [23]. With the cost of RNA-Seq continuing to drop in the future, our RNA editing-based classification will become more practical and gain more widespread adoption by laboratories and clinics. Second, RNA editing-based classification has the potential to help understand mechanisms driving gliomagenesis and indicate how the tumor could behave in the future. Many of the editing sites used in our models could serve as prognostic markers. For example, chr6:159679878 (one of the sites used to predict 1p/19q codeletion) has prognostic value for LGG patients. Patients with higher level of editing at chr6:159,679,878 have worse OS and progression free interval than those with lower editing (log-rank test: *p* < 0.0001; Fig. 4). This site resides in 3’UTR of the gene *mitochondria-*localized manganese superoxide dismutase (MnSOD/SOD2). SOD2 has both tumor promoting and suppressing functions in cancer [24]. It has been suggested that the dichotomous function of *SOD2* results from the context-dependent regulation of *SOD2* during different stages of tumor development [24]. The dynamic nature of RNA editing might play a role in the temporal regulation of *SOD2* during cancer development, although further investigation is needed.

Although our model classifies gliomas using only RNA-seq data, it can be adjusted easily to include other -omic data (such as methylation chip and/or exome sequencing). Several studies have shown that DNA methylation is able to cluster/classify brain tumors. The beta value obtained from the methylation chip is between [0,1] (same as the value of RNA editing). It is straightforward to include methylation data in our models and likely to increase the performance. We also developed models to classify patients into one of three groups simultaneously. However, SVM and AB had lower AUCs in predicting IDH mut-non-codel (See Additional file 1: Figure S1).

Some limitations of the study should be considered. First, we did not have an independent validation cohort to assess the performance of our classifiers. Second, our classifiers may only apply to adult gliomas and its performance on children brain tumors requires further investigation. Furthermore, our models are not able to distinguish grade II and grade III (anaplastic) astrocytoma, which are still listed in WHO 2016 classification guidelines.

## Conclusions

In summary, our results reveal the clinical utility of RNA editing in glioma classification. Our prediction models provide standardized, accurate, reproducible and objective classification of gliomas. Our model is not only useful for clinical decision-making, but also able to identify editing events that have the potential to serve as biomarkers and therapeutic targets in glioma management and treatment.

## Methods

### Data collection

We downloaded The Cancer Genome Atlas (TCGA) LGG (low grade glioma) and GBM (glioblastoma multiforme) RNA-Seq bam files (v.2016) and clinical information from Genomic Data Commons (GDC [18];). We selected tumors that have both RNA-Seq bam files and annotation of *IDH* mutation and 1p/19q codeletion available, resulting in 638 samples (496 LGG and 142 GBM). *IDH* mutation is defined as carrying any type of known *IDH1* or *IDH2* mutation. The status of *IDH* mutation and 1p/19q codeletion of each sample was downloaded from [25].

### Identification of RNA editing events in gliomas

Similar to Peng et al. [26], we used REDItools [27] with default settings to detect editing events on sites reported in REDIportal [19], currently the most comprehensive A-to-I RNA editing database. Hyper-edited reads were detected according to Porath et al. [28] and only reads covering sites in REDIportal were included. For each sample, both total editing level and site-specific editing level were calculated. Total editing level was calculated by dividing the number of reads with the edited G nucleotide by total number of A + G reads of the sample. Site-specific editing level was calculated by dividing the number of covering edited G reads by the number of covering A + G reads of an editing site. Because DNA changes could result in misidentification of RNA editing events, we downloaded somatic mutation data from GDC and germline variant data from the TCGA Pan-Cancer analysis project [29] and GDC legacy archive. Editing events overlapping with sample-specific somatic mutations or germline variants were excluded.

### Selection of discriminative editing sites

We focused on sites that have better discriminative power for classifying gliomas by dividing sites into three types (based on the number of covering edited G and A + G reads): (1) Type I: (a) covered by ≥3 edited G reads and ≥ 10 A + G reads; and (b) editing frequency (edited G reads / A + G reads) is significantly greater than 0.1% (binomial test with FDR < 0.05); (2) Type II: covered by ≥10 reads but (a) < 3 edited G reads or (b) editing frequency is not significant greater than 0.1% (binomial test); and (3) Type III:: covered by < 10 reads. We first removed sites that were Type III in > 25% samples, resulting in 65,428 sites. We next selected sites with larger editing variability among samples. Median absolute deviation (MAD), defined as the median of the absolute deviations from the data’s median, is often used to measure data variability. To include more sites, here we defined MAD_3Q_ as the median of the absolute deviations from the data’s third quantile. We required sites to be Type I in at least 25% samples and with MAD_3Q_ > 0, resulting in 10,642 sites as starting features in our prediction models.

### Annotation of editing sites

Gene structure (RefSeq) and variant (dbSNP, ExAc, and gnomAD) information of editing sites were derived from ANNOVAR (2018 Jul 08) [20].

### Machine learning algorithms, ten-fold cross-validation, evaluation measures, and feature selection

Three supervised learning algorithms including support vector machines (SVM), random forest (RF) and AdaBoost (AB) were used in this study to predict the status of *IDH* mutation and 1p/19q codeletion. SVM is popular because of its accuracy and less usage of computational resource. We selected linear kernel because other kernel functions did not perform better. RF, an ensemble method, first fits a number of decision tree classifiers on various sub-samples of the dataset and then uses averaging to improve accuracy and control over-fitting. AdaBoost (short for “Adaptive Boosting”; AB) is another ensemble learning method, which aims to create a strong classifier from a number of weak classifiers. Python’s scikit-learn (with default parameters) was used to build the above classifiers.

Our models were evaluated using 10-fold cross-validation, which is widely used when sample size is limited and provides a more accurate estimate of prediction performance. The process started from randomly separating the whole dataset into 10 groups with equal size. In each validation, nine groups were used to train the model (i.e., training group) and one group (i.e., test group) was used to evaluate the performance. Accuracy (ACC), sensitivity (SEN), specificity (SPE), and area under the receiver operating characteristics curve (AUC) were used to assess model performance. ACC was calculated as the sum of correct predictions (TP+ TN) divided by total number of predictions (TP + FP + TN + FN), where TP, FP, TN, and FN, respectively, represents true positives, false positives, true negatives, and false negatives. SEN was calculated by TP/(TP + FN) and SPE was calculated by TN/(TN + FP). ROC curves were plotted using SEN and 1-SPE under different cutoff points. The above validation process was repeated 10 times and thus, the whole dataset was completely assessed. We also performed feature selection within each fold for both *IDH* mutation and 1p/19q codeletion classifiers to avoid overfitting and possibly improve prediction performance.

### Survival analysis

Kaplan-Meier (KM) method was used to analyze the association between editing level and overall survival (OS) and progression free interval (PFI). Log-rank test was used to examine statistical significance. Seven and three samples, respectively, were not included in the OS and PFI plots because of lack of data or more than 10 years.

## Supplementary information

**Additional file 1: Figure S1.** ROC plots for predicting isocitrate dehydrogenase (IDH) mutation and chromosome 1p/19q codeletion simultaneously. We trained SVM, RF, and AB with feature selection within each fold (10-fold cross validation) to classify patients into one of three groups simultaneously. a. IDH wt. b. IDH mut-codel. c. IDH mut-non-codel.

## Data Availability

The datasets used and/or analyzed during the current study are available from the corresponding author on reasonable request.

## Abbreviations

3’UTR: 3′ untranslated regions
AB: AdaBoost
ACC: Accuracy
AUC: Area under the ROC curve
FISH: Fluorescent in situ hybridization
GBM: Glioblastoma multiforme
IDH mut-codel: Samples with both IDH mutation and chromosome 1p/19q codeletion
IDH mut-non-codel: Samples with only IDH mutation (no 1p/19q codeletion)
IDH wt: Samples with wildtype isocitrate dehydrogenase (*IDH*)
IDH: Isocitrate dehydrogenase
IHC: Immunohistochemistry
KM: Kaplan-Meier
LGG: Low grade glioma
OS: Overall survival
PFI: Progression free interval
RF: Random forest
ROC: Receiver operating characteristic
SEN: Sensitivity
SPE: Specificity
SVM: Support vector machines
TCGA: The cancer genome atlas
