# Supplementary figures and images for: RNA editing-based classification of diffuse gliomas: predicting isocitrate dehydrogenase mutation and chromosome 1p/19q codeletion
Source: BMC Bioinformatics. 2019 Dec 24;20(Suppl 19):659. doi: 10.1186/s12859-019-3236-0 (PMC6929429; doi:10.1186/s12859-019-3236-0)

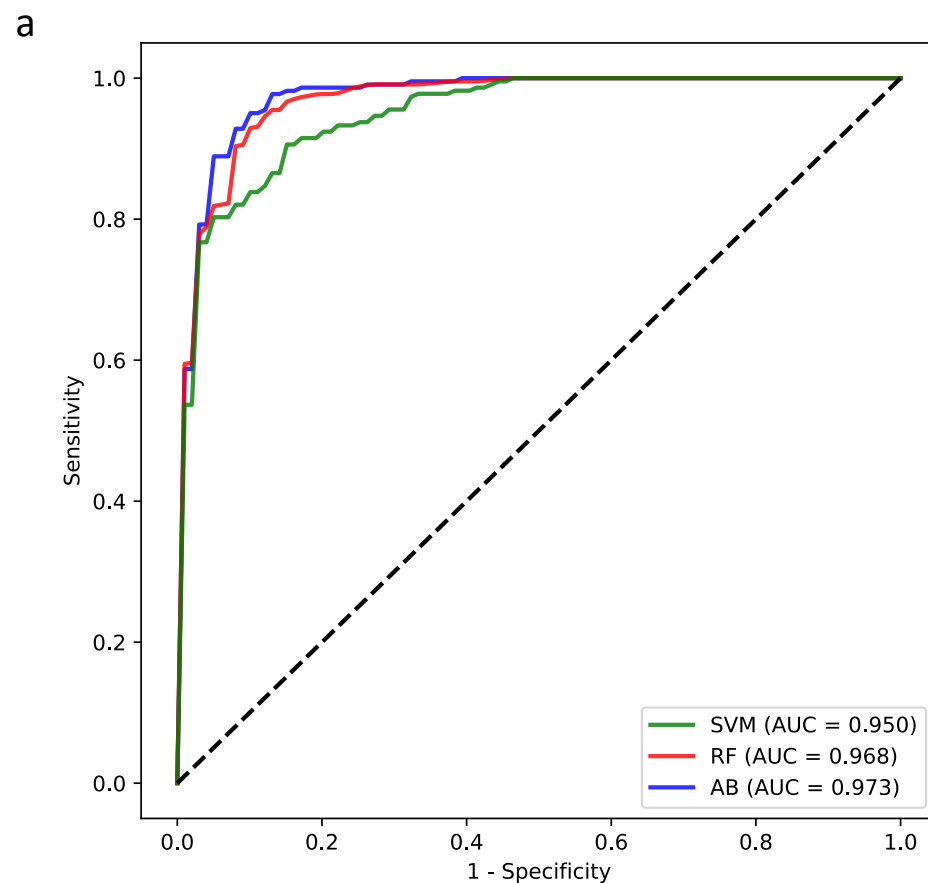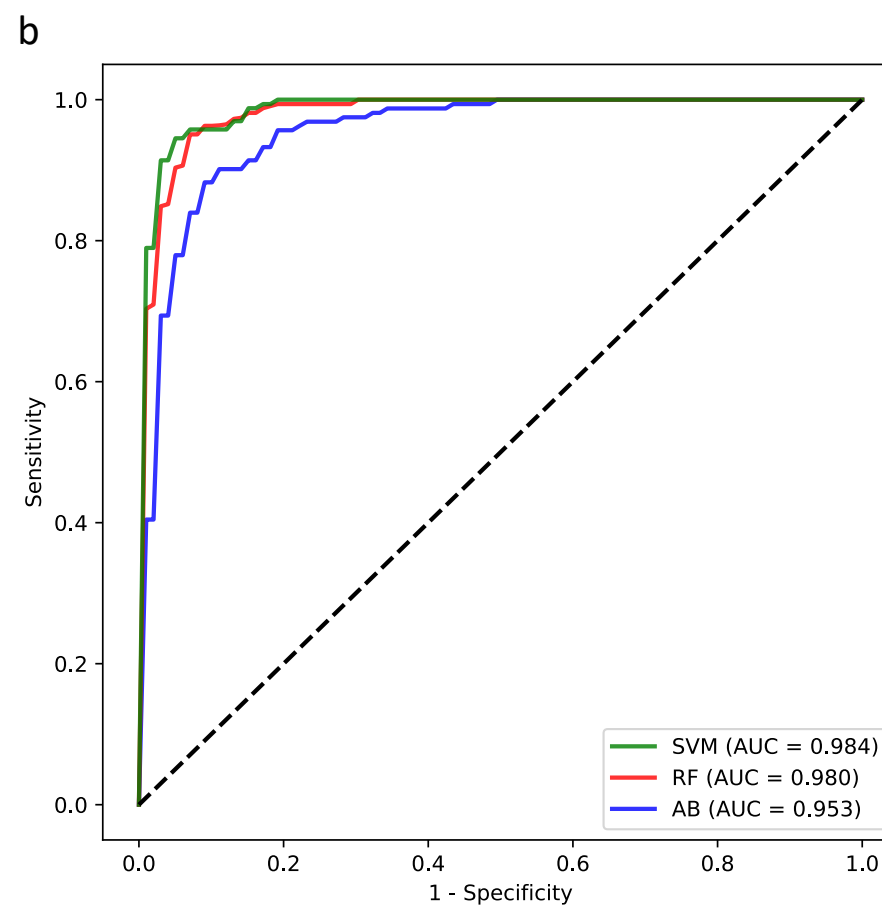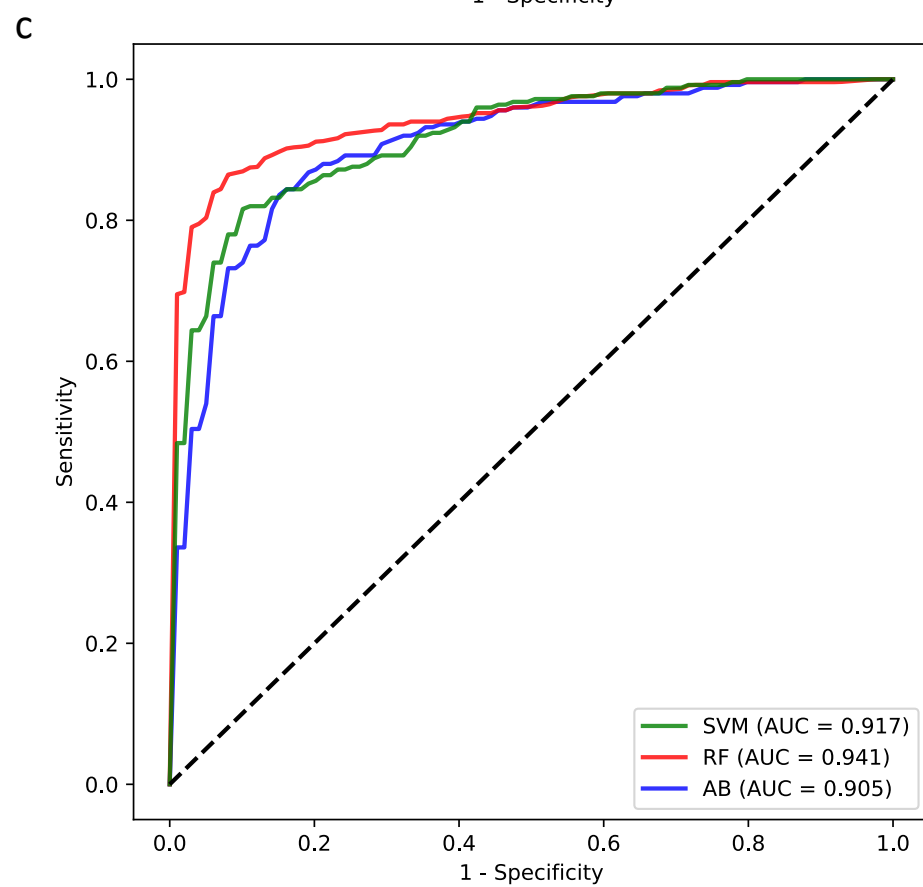

Supplement: Supplementary file 1 — Additional file 1: Figure S1. ROC plots for predicting isocitrate dehydrogenase (IDH) mutation and chromosome 1p/19q codeletion simultaneously. We trained SVM, RF, and AB with feature selection within each fold (10-fold cross validation) to classify patients into one of three groups simultaneously. a. IDH wt. b. IDH mut-codel. c. IDH mut-non-codel. [file 12859_2019_3236_MOESM1_ESM.pdf]
